# Supplementary material for: A method for rapid machine learning development for data mining with doctor-in-the-loop
Source: PLoS One. 2023 May 10;18(5):e0284965. doi: 10.1371/journal.pone.0284965 (PMC10171605; doi:10.1371/journal.pone.0284965)
Supplement: Attachment — Submitted filename: Response to reviewers.docx [file pone.0284965.s001.docx]

Response to reviewers

PONE-D-22-29006: A method for rapid machine learning development for data mining with Doctor-In-The-Loop

| 1. Please ensure that your manuscript meets PLOS ONE's style requirements, including those for file naming. The PLOS ONE style templates can be found at  <https://journals.plos.org/plosone/s/file?id=wjVg/PLOSOne_formatting_sample_main_body.pdf> and  <https://journals.plos.org/plosone/s/file?id=ba62/PLOSOne_formatting_sample_title_authors_affiliations.pdf> | The manuscript has been reformatted according to the style guide. |
| --- | --- |
| 2. In your Data Availability statement, you have not specified where the minimal data set underlying the results described in your manuscript can be found. PLOS defines a study's minimal data set as the underlying data used to reach the conclusions drawn in the manuscript and any additional data required to replicate the reported study findings in their entirety. All PLOS journals require that the minimal data set be made fully available. For more information about our data policy, please see<http://journals.plos.org/plosone/s/data-availability>.  Upon re-submitting your revised manuscript, please upload your study’s minimal underlying data set as either Supporting Information files or to a stable, public repository and include the relevant URLs, DOIs, or accession numbers within your revised cover letter. For a list of acceptable repositories, please see <http://journals.plos.org/plosone/s/data-availability#loc-recommended-repositories>. Any potentially identifying patient information must be fully anonymized.  Important: If there are ethical or legal restrictions to sharing your data publicly, please explain these restrictions in detail. Please see our guidelines for more information on what we consider unacceptable restrictions to publicly sharing data: <http://journals.plos.org/plosone/s/data-availability#loc-unacceptable-data-access-restrictions>. Note that it is not acceptable for the authors to be the sole named individuals responsible for ensuring data access.  We will update your Data Availability statement to reflect the information you provide in your cover letter. | **The jurisdiction of the Northern Territory is very unique, with small and remote communities where specific incidents of death or retrieval are clearly identifiable even by date alone.**  **Both datasets (mortality and aeromedical retrieval) contain sensitive personal data, including date, location and cause (of death or aeromedical retrieval). This information is integral to train the algorithm and is essential to replicate our method.**  **The study was performed on this data with ethics approval from the NT Department of Health / Menzies School of Health Research Human Research Ethics Committee (HREC) (reference number 2021-4056).**  **This data was provided by data custodians on the basis of this HREC approval from:**   - **Mortality data provided by the Australian Births, Deaths and Marriages (BDM) Registry.** - **Aeromedical retrieval data provided by the Royal Flying Doctor Service and Careflight Australia (NT) services.**   **Use of these data sets can only be accessed with appropriate HREC approval.**  **The authors will provide a letter of support to the NT Department of Health / Menzies School of Health Research HREC (with subsequent confirmation received from data custodians) to any research or academic team wishing to replicate our method using this data. The HREC has confirmed support of this approach and will facilitate rapid review of such an application. With this external teams’ formal HREC approval, our team will provide the original raw data for further analysis under the proviso that all data transactions conform with NT Department of Health / Menzies School of Health Research HREC requirements.** |
| The authors present a decision-making tool that involves clinical experts to participate in the training of off-the-shelf machine learning algorithms aimed at classifying free-text medical notes. The article is well written and easy to follow, and the tool presented seems to offer a means for clinicians to get involved in the crafting of the tool and in improving its performance, which may eventually help with the clinical practicality. However, the proposed framework can hardly be claimed as original work as it is essentially an active learning approach applied to the problem of free-text classification. The tool could in a way be regarded as novel, but not a research novelty. The authors need to clarify and support what they propose to be truly original.  Reviewer #1: While the manuscript presents a system of potential clinical application, it lacks originality. The proposed training/evaluation protocol has extensively been used and its merit in improving the performance of machine learning algorithms has been thoroughly discussed in many published studies. Additionally, the idea of involving human experts in the training phase of a machine learning based decision-making tool is not new and links to Active Learning. It is understood that such a tool may have a clinical practicality and involving clinicians in its development may contribute to its performance improvement, it remains unclear however if the originality of the work is the tool itself or the framework to develop it. | **The originality of our paper is that it demonstrates the ready use of open source AutoML frameworks (in our case ML.NET), which in essence is proof of the democratisation of ML techniques. We have now highlighted this in the discussion (paragraph 2, Discussion).**  **The result is that it is now possible to use ML to assist clinical research without postgraduate level ML expertise. The implications of this are significant. Principally, it brings the utility of ML in processing large datasets to laboratories that may not have sufficient funding to employ ML specialists. A fundamental understanding of statistics is still required, and we assume that clinical researchers have this.**    **The method we describe differs from typical active learning in that there was no automation of selection of new datapoints to label.  We demonstrate that it is both sufficient and efficient to use the real intelligence of humans (in our case; Doctors) to determine which additional cases to label.**    **Of note, we chose to demonstrate that efficiency was reproducible across two different data sets and categorisation tasks. We did not do a direct comparison to any of the myriad of existing active learning techniques, as it was not conceived as an attempt to advance the field of active learning from a computer science perspective.  Similarly, we elected not to include in the manuscript a lengthy review of active learning and Human-In-The-Loop strategies as the intended audience is scientists who have a need for categorization of large datasets but don’t come from a computer science background. For these reasons we also chose to avoid Deep Learning.  We may have achieved superior results using a transformer such as BERT, however this would add significant complexity to the software development step – defeating the purpose of creating an easily reproducible method that doesn’t require advanced ML and computer science skills.**    **In essence, the manuscript is a description of how ML can be implemented quickly, cheaply, achieve high accuracy using the existing resources of a typical research laboratory.  This goes to the Utility requirement of PLOS ONE’s submission guidelines, the manuscript presents experimental data demonstrating the Validation requirement. The tool was developed using open-source libraries and the manuscript provides sufficient detail for the tool to be replicated in any programming framework capable of ML. This ensures Availability of the method.  As for Novelty, our reviews of the literature  did not find this combination of techniques previously reported.** |
|  |  |
